# A putative Chondroprotective role for IL-1β and MPO in herbal treatment of experimental osteoarthritis

**DOI:** 10.1186/s12906-017-2002-y

**Published:** 2017-11-22

**Authors:** Nora M. Aborehab, Mahitab H. El Bishbishy, Abeer Refaiy, Nermien E. Waly

**Affiliations:** 1 Department of Biochemistry, Faculty of Pharmacy, MSA University, Giza, 11787 Egypt; 2 Department of Pharmacognosy, Faculty of Pharmacy, MSA University, Giza, 11787 Egypt; 3 Department of Pathology Faculty of Medicine, Assiut University, Assiut, 71515 Egypt; 4 Department of Physiology, Faculty of Medicine, Helwan University, Cairo, 11795, Egypt

**Keywords:** Ginger, Curcumin, Osteoarthritis, IL-1β, COMP, HA, MPO, MDA

## Abstract

**Background:**

Herbal treatment may have a chondroprotective and therapeutic effect on Osteoarthritis (OA). We investigated the mechanism of action of ginger and curcumin rhizomes cultivated in Egypt in treatment of OA in rat model.

**Methods:**

Thirty-five albino rats were intra-articularly injected with Monosodium Iodoacetate in the knee joint. Ginger and curcumin was orally administered at doses of 200 and 400 mg/kg (F200 and F400). Serum levels of cartilage oligomeric matrix protein (COMP), hyaluronic acid (HA), malondialdehyde (MDA), myeloperoxidase (MPO), Interleukin-1 beta (IL-1β) and superoxide dismutase activity (SOD) were measured using ELISA. The composition of the herbal formula hydro-ethanolic extract was characterized using UPLC-ESI-MS. Histopathological changes in injected joints was examined using routine histopathology. Statistical analysis was performed using one-way ANOVA.

**Results:**

Serum levels of COMP, HA, MPO, MDA, and IL-1β were significantly decreased in F 200, F 400 and V groups when compared to OA group (*P* value <0.0001). On the other hand SOD levels were significantly elevated in treated groups compared to OA groups (P value <0.0001).

**Conclusions:**

The ginger/curcumin at 1:1 had chondroprotective effect via anti-inflammatory and antioxidant effect in rat OA model. Further pharmacological and clinical studies are needed to evaluate this effect.

## Background

Osteoarthritis (OA) is a common degenerative joint disease in the elderly and a leading cause of physical disability worldwide [1]. It mainly affects the weight– bearing joints, such as hips and knees [2]. OA is a degenerative disease characterized by structural changes of the knee joint including articular cartilage erosion, synovitis and remodeling of subchondral bone [3]. Patients affected by OA experience pain that worsens with motion, exercise and, with pathology progression, also at rest [4]. It was proposed that OA is the result of both mechanical and biological changes, causing loss of the normal homeostatic mechanism occurring between catabolic and anabolic processes in the articular cartilage. Chondrocyte in the mature articular cartilage is the only type of cell that is capable of production and maintenance of the extracellular matrix. Therefore any reduction in chondrocyte density will contribute to the development of OA [5]. To date, there is no effective pharmacological treatment for OA and the end stage of disease usually requires joint replacement surgery [6, 7].

A monosodium Iodoacetate (MIA) induced OA model is well described in rats especially in terms of the pathological progression of the disease. It triggers changes similar to the histological and pathophysiological features of that in human OA. The mechanism of action of MIA has been attributed to inhibition of glyceraldehyde-3-phosphate dehydrogenase. This disrupts the glucose metabolism of chondrocytes, leading to reactive oxygen species (ROS) production and caspase activation, and further to the catabolism of cartilage matrix and cell death, that can be detected both in vivo and in vitro. It therefore targets the avascular cartilage and causes chondrocyte death, fragmentation of cartilage and exposure of subchondral tissue. In addition to evoking cartilage degradation, MIA induces an acute inflammation, which is associated with increased expression of pro-inflammatory factors such as IL-1β IL-6, IL-15, COX-2 and metalloproteinase [8–10].

Since the pharmacologic treatment has several limitations and is not always effective in the management of OA, researchers have been striving to find alternative therapeutic agents. Herbs and extracts present a potential hope for treatment of many diseases. The use of tomato and broccoli extracts has been shown to have beneficial effect in the treatment of obesity both in rats and humans [11–13].

Ginger, *Zingiber officinale Rosc.,* rhizomes have been shown to contain a large amount of zerurnbone [14]. Zerurnbone has been shown to have anticancer and antioxidant actions [5]. In addition several studies has shown that ginger possesses medicinal effects in animals. Its ethanol extract had hypoglycemic and anti-inflammatory effects in rats [15]. Another study has shown that ginger protects against ulcer development in albino rats [16]. Ginger also has been shown to exert chondroprotective effects in an experimental model of OA in rats [5]. In addition it is safe to use as the LD_50_ of ginger extract was is up to 1 g/kg [17].

*Curcuma longa* L., *Zingiberaceae* (turmeric) is a herb that is used in the Fareast folk medicine for the treatment of biliary disorders among other diseases [18]. Its extract has been shown to have beneficial effects in the treatment of OA through lowering oxidative stress and reducing inflammation [19, 20]. It has also been shown to have protective effects against rheumatoid arthritis in rat model [21]. Furthermore, ginger has no toxic effects when administered up to 5 g/kg in rats which provides wide safety margin of testing for its beneficial effects [22].

The aim of this study was to characterize the composition of the ginger and turmeric herbal formula hydro-ethanolic extract evaluate the possible chondro-protective effect of a mixed formula of both herbs in MIA induced OA rat model. In addition we investigated the possible mechanism of action of the herbal formula and examine its histopatholgical effect compared it to standard therapeutic agents.

## Methods

### Animals

Thirty-five male albino rats, weighing 200 ± 30 g at the start of the experiments were used. Prior to the initiation of the studies, the animals were randomized and assigned to treatment groups. Four rats were housed per cage (size 26 × 41 cm) and placed in the experimental room for acclimatization 24 h before any procedure was carried out. The animals were fed with standard laboratory diet and tap water ad libitum, and kept in an air-conditioned animal room at 23 ± 1 °C with a 12 h light/dark cycle.

Animal care and handling was performed in conformance with approved protocols of Cairo University and Egyptian Community guidelines for animal care. Research Ethics Committee at MSA School of Pharmacy approved the protocol of this study.

### Monosodium iodoacetate injection

A single intra-articular (i.a.) injection of 2 mg of MIA (Sigma-Aldrich, Egypt) was performed through the infra-patellar ligament into the joint space of the right knee of lightly anesthetized rats (3% isoflurane in O2 at 1.5 l/min) in a total volume of 50 μl saline according to methodology of Nagase et al., 2012 [8].

### Plant material

Fresh rhizomes of *Z. officinale* Rosc., and *C. longa* L., Zingiberaceae were purchased from the local market, Giza, Egypt during 2015/2016. The taxonomical identity was kindly verified by Dr. Mohamed El Gebaly, National Research Center, Giza, Egypt. The plant samples were air dried in the absence of direct sunlight and ground just before extraction.

### Herbal formula preparation

The plant material was separately grinded then mixed together in a 1:1 ratio, macerated in 70% ethanol till exhaustion. The hydro-ethanolic extracts were concentrated under reduced pressure and kept in tightly closed amber glass containers for LC-ESI-MS and biological analysis. A voucher specimen (RS 0019) was deposited in the herbarium of faculty of Pharmacy, MSA University.

### Experimental groups

Rats were randomly allocated into five groups of seven animals each.

Group 1: Normal control group injected (i.a) by 50 μl saline in the right knee.

Group 2 (OA): rats injected (i.a) of 2 mg of MIA in a total volume of 50 μl saline in the right knee.

Group 3 (F 200): Osteoarthritic rats treated with Herbal formula 200 mg/kg/day, orally for 1 month.

Group 4 (F 400): Osteoarthritic rats treated with Herbal formula 400 mg/kg/day, orally. For 1 month.

Group 5 (V): Osteoarthritic rats treated with Voltaren 30 mg/kg/day, orally for 1 month.

Doses were selected based on previous published results of toxicity studies in similar species [17, 22].

### Drugs

Voltaren (Novartis, Egypt) were dissolved in distilled water and administered orally.

### Chemicals

Ethanol was of HPLC grade and purchased from Sigma–Aldrich (Steinheim, Germany).

### Blood samples and biochemical analysis

At the end of the study, rats were fasted overnight, anesthetized with thiopental sodium (50 mg/kg) [23] and blood samples were collected in the morning (5 ml per rat). Blood samples were centrifuged at 3000 rpm for 15 min after 30 min of collection and stored at −80 °C until analyzed. Serum cartilage oligomeric matrix protein (COMP), Hyaluronic acid (HA), Interleukin-1β (IL-1), Myeloperoxidase (MPO), Superoxide dismutase (SOD) were measured using the corresponding rat enzyme immunoassay kits (COMP: Cusabio Biotech Co, China), (HA: Uscn Life Science Inc. Wuhan, China), (IL-1: Koma Biotech Inc., Korea), (MPO: Hycult Biotech, Netherland), and (SOD: Cusabio Biotech Co, China) according to manufactured instructions. Serum Malondialdehyde (MDA): Cell Biolabs, Inc., USA was determined quantitatively using spectrophotometric method.

### Histopathological examination

Anesthetized rats (thiopental sodium (50 mg/kg) [23])were rapidly decapitated; knee joints were dissected, rinsed in saline. Specimens were fixed in 10% formalin and then joints were decalcified in nitric oxide for 4 days, routinely processed and embedded in paraffin. Five microns sections were cut and stained with hematoxylin and Eosin (H&E). Histopathological evaluation were done according to Osteoarthritis Research Society International (OARSI) cartilage OA grading system [24].

### UPLC-ESI–MS apparatus

The analysis was performed on a MS QQQ mass spectrometer coupled to Aglient 6420 series UPLC system (Agilent Technologies, Waldbronn, Germany), equipped with a 1290 ultra-performance auto sampler, 1290 infinity Quad pump, and 6420 triple quadrupole detector. Chromatographic separation was performed on a Acquity UPLC® HSS T3 (150 mm × 2.1 mm i.d.; 1.8 μm) column (Waters, USA).

### Identification of the major compounds

Mobile phase consisted of two solvents, (A) 10% acetonitrile and (B) water. The separation was performed using gradient elution, from 15% to 90% A at 40 °C at a flow rate of 0.35 mL/min. Sample injection volume was 5 μL. The ionization technique was electrospray ionization. Spectra were recorded in negative ion mode between m/z 50 and 1000 with capillary voltage, 4500 V and heated dry nitrogen gas temperature, 325 °C and flow rate 9 l/min, the gas flow to the nebulizer was set at pressure 60 psi.

### Statistical analyses

All data were expressed as mean ± SEM and analyzed using Prism program version 6. For all parameters, comparisons among groups (*N* = 7) were carried out using one-way analysis of variance (ANOVA) followed by Bonferroni’s multiple comparisons test. All *P* values reported are two-tailed and *P ‹ 0.05* was set as the level of significance.

## Results

### Effect of herbal formula treatment on blood cartilage oligomeric matrix protein (COMP)

**Fig. 1:**
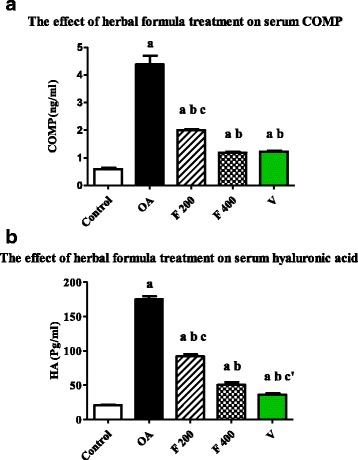
**a** Serum level of COMP (ng/ml) in the experimental groups. Herbal formula treatment decreased serum level of COMP in the osteoarthritic rats at the end of 1 month treatment; C = control; OA = osteoarthritis; F 200 = herbal formula 200 mg/kg; F 400 = herbal formula 400 mg/kg; V = Voltaren. Results were expressed as mean ± SEM and analyzed using one-way ANOVA followed by Bonferroni’s post hoc test a **=** Significant from control at *P* < 0.0001, b **=** Significant from OA at *P* < 0.0001, c = Significant from F 400 at *P* < 0.0001. **b** Serum level of HA (Pg/ml) in the experimental groups. Herbal formula treatment decreased serum level of HA in the osteoarthritic rats at the end of 1 month treatment; C = control; OA = osteoarthritis; F 200 = herbal formula 200 mg/kg; F 400 = herbal formula 400 mg/kg; V = Voltaren. Results were expressed as mean ± SEM and analyzed using one-way ANOVA followed by Bonferroni’s post hoc test a **=** Significant from control at *P* < 0.0001, b **=** Significant from OA at *P* < 0.0001, c = Significant from F 400 at *P* < 0.0001, c’ = Significant from F 400 at *P* < 0.005

Mean serum level of COMP was significantly increased in OA group compared to the control group (*P* value <0.0001). On the other hand, the mean serum level of COMP was significantly decreased in F 200, F 400 and V groups when compared to OA group (P value <0.0001). The mean serum level of COMP was significantly decreased in F 400 group compared to F 200 group (*P* value <0.0001). Non-significant difference of COMP serum level was found between F 400 group and V group (Figure 1a).

### Effect of herbal formula on blood hyaluronic acid (HA)

Mean serum level of HA was significantly increased in OA group compared to the control group (*P* value <0.0001). The mean serum level of HA was significantly decreased in F 200, F 400 and V groups when compared to OA group (*P* value <0.0001). The mean serum level of HA was significantly decreased in F 400 group compared to F 200 group (*P* value <0.0001). Also a significant difference was found between V and F 400 groups (*P* value <0.005) (Fig. 1b).

### Effect of herbal formula on blood myeloperoxidase (MPO)

**Fig. 2:**
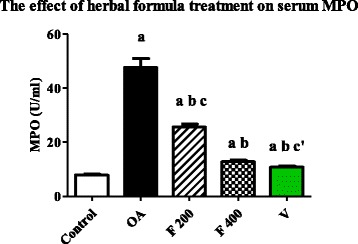
Serum level of MPO (U/ml) in the experimental groups. Herbal formula treatment decreased serum level of MPO in the osteoarthritic rats at the end of 1 month treatment; C = control; OA = osteoarthritis; F 200 = herbal formula 200 mg/kg; F 400 = herbal formula 400 mg/kg; V = Voltaren. Results were expressed as mean ± SEM and analyzed using one-way ANOVA followed by Bonferroni’s post hoc test a **=** Significant from control at *P* < 0.0001, b **=** Significant from OA at *P* < 0.0001, c = Significant from F 400 at *P* < 0.0001, c’ = Significant from F 400 at *P* < 0.01

Mean serum level of MPO was significantly increased in OA group compared to the control group (*P* value <0.0001). The mean serum level of MPO was significantly decreased in F 200, F 400 and V groups when compared to OA group (*P* value <0.0001). The mean serum level of MPO was significantly decreased in F 400 group compared to F 200 group (*P* value <0.0001). A significant difference was found between V and F 400 groups (P value <0.01) (Fig. 2).

### Effect of herbal formula on blood Interleukin-1 beta (IL-1β)

**Fig. 3:**
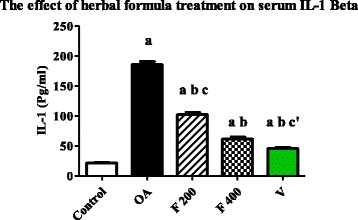
Serum level of IL-1 Beta (Pg/ml) in the experimental groups. Herbal formula treatment decreased serum level of IL-1 Beta in the osteoarthritic rats at the end of 1 month treatment; C = control; OA = osteoarthritis; F 200 = herbal formula 200 mg/kg; F 400 = herbal formula 400 mg/kg; V = Voltaren. Results were expressed as mean ± SEM and analyzed using one-way ANOVA followed by Bonferroni’s post hoc test, a **=** Significant from control at *P* < 0.0001, b **=** Significant from OA at *P* < 0.0001, c = Significant from F 400 at *P* < 0.0001, c’ = Significant from F 400 at *P* < 0.002

Mean serum level of IL-1β was significantly increased in OA group compared to the control group (*P* value <0.0001). The mean serum level of IL-1β was significantly decreased in F 200, F 400 and V groups when compared to OA group (*P* value <0.0001). The mean serum level of IL-1β was significantly decreased in F 400 group compared to F 200 group (*P* value <0.0001). A significant difference was found between V and F 400 groups (*P* value <0.002) (Fig. 3).

### Effect of herbal formula on blood malondialdehyde (MDA) and superoxide dismutase (SOD)

**Fig. 4:**
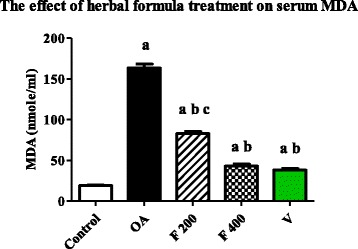
Serum level of MDA (nmole/ml) in the experimental groups. Herbal formula treatment decreased serum level of MDA in the osteoarthritic rats at the end of 1 month treatment; C = control; OA = osteoarthritis; F 200 = herbal formula 200 mg/kg; F 400 = herbal formula 400 mg/kg; V = Voltaren. Results were expressed as mean ± SEM and analyzed using one-way ANOVA followed by Bonferroni’s post hoc test, a **=** Significant from control at *P* < 0.0001, b **=** Significant from OA at *P* < 0.0001, c = Significant from F 400 at *P* < 0.0001

Mean serum level of MDA was significantly increased in OA group compared to the control group (*P* value <0.0001). The mean serum level of MDA was significantly decreased in F 200, F 400 and V groups when compared to OA group (*P* value <0.0001). The mean serum level of MDA was significantly decreased in F 400 group compared to F 200 group (*P* value <0.0001). While non-significant difference was found between F 400 and V groups. (Fig. 4).

**Fig. 5:**
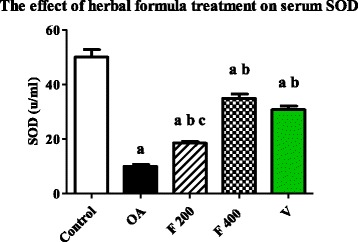
Serum level of SOD (U/ml) in the experimental groups. Herbal formula treatment increased serum level of SOD in the osteoarthritic rats at the end of 1 month treatment; C = control; OA = osteoarthritis; F 200 = herbal formula 200 mg/kg; F 400 = herbal formula 400 mg/kg; V = Voltaren. Results were expressed as mean ± SEM and analyzed using one-way ANOVA followed by Bonferroni’s post hoc test, a **=** Significant from control at *P* < 0.0001, b **=** Significant from OA at *P* < 0.0001, c = Significant from F 400 at *P* < 0.0001

The mean serum level of SOD was significantly decreased in OA group compared to the control group, while it was increased in F 200, F 400 and V groups when compared to OA group (*P* value <0.0001). The mean serum level of SOD was significantly increased in F 400 group compared to F 200 group (P value <0.0001) and non-significant difference was found between F 400 and V groups. (Fig. 5).

### UPLC-ESI-MS characterization of the herbal formula

UPLC-ESI-MS analysis of the hydro-ethanolic extract of the herbal formula demonstrated the presence of several gingerol derivatives in addition to curcumin and dihydro-curcumin as previously reported in different ginger and turmeric extracts [25–29]. In total, 11 compounds belonging to different classes were tentatively identified by UPLC–MS. Data of peaks identification, retention times, and negative ion mode tentative identification of major compounds detected is presented in Table 1.

**Table 1:** Chromatographic, mass spectral characteristics and tentative identification of compounds in the herbal formula by UPLC-ESI(−ve)-MS

Compound	T_r_ (min)	Tentative Identification	Molecular Weight	(−)-ESI-MS (*m/z*)	Reference(s)
1	2.07	10- gingerdiol	352	351 [M-H]-	Tanaka et al. 2015 [28]
2	7.76	Acetoxy-10-gingerol	392	391 [M-H]-	H. Jiang et al. 2005 [25]
3	9.43	13-Paradol	376	375 [M-H]-	Tanaka et al. 2015 [28]
4	14.91	1- Dehydro-12- gingerdione	374	373 [M-H]-	H. Jiang et al. 2005 [25]
5	16.09	Methyl-6-gingerol	308	307 [M-H]-	L. B., Ahui M. et al. 2013 [27]
6	19.24	7- Paradol	292	291 [M-H]-	Tanaka et al. 2015 [28]
7	19.86	Curcumin	368	367 [M-H]-	Herebian et al. 2009 [29]
8	22.17	Methyl diacetoxy-6-gingerdiol	394	393 [M-H]-	Tanaka et al. 2015 [28]
9	22.88	10-gingerol	350	349 [M-H]-	H. Jiang et al. 2005 [25] TAO et al. 2009 [26] L. B., Ahui M. et al. 2013 [27]
10	23.6	6-Paradol	278	277 [M-H]-	TAO et al. 2009 [26] Tanaka et al. 2015 [25]
11	26.29	Dihydrocurumin	370	369 [M-H]-	Herebian et al. 2009 [29]

### Histopathological changes associated with glucosamine treatment

Sections from the control group (C) showed normal histological appearance and proteoglycan content of the cartilage as judged by the preserved integrity of the articular surface, normal orientation and distribution of chondrocytes and the preservation of superficial layer. The OA group showed features of osteoarthritis grade 5 (ORASI grading system) recognized by denudation and erosion of the cartilage with reparative fibrotic changes (clefting, chondrocytes degeneration, calcification, fibrosis and matrix changes).

**Fig. 6:**
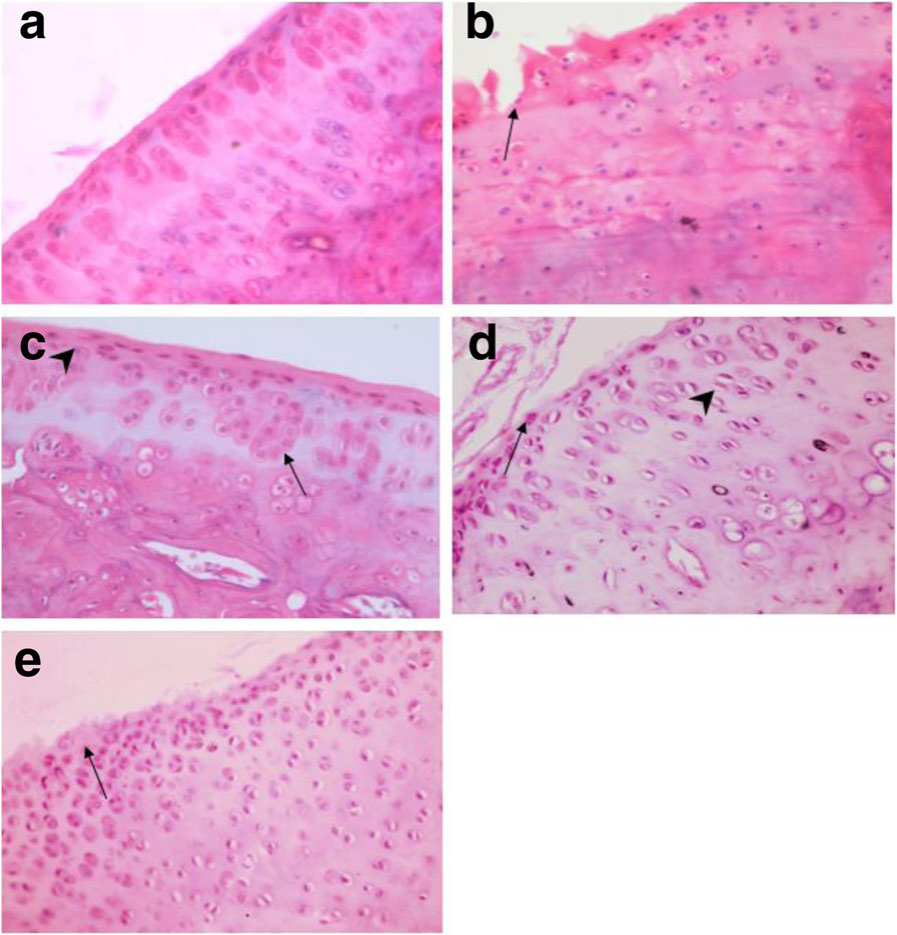
Photomicrograph of **a** control group showing normal chondrocytes shape and orientation. **b** From osteoarthritis group showing denudation, erosion, clefting (arrow) and degeneration of chondrocytes. **c** F400 group showing superficial fibrillation (arrow head) with more or less normal orientation and some edema (arrow). **d** From F200 group superficial fibrillation (arrow), proliferation and clustering of chondrocytes, (arrow head) cracking and simple fissures. **e** From Voltarin treated group showing Loss of superficial layer, chondrocytes necrosis and simple fissures (arrow) H&Ex400

Other groups showed different grades of OA according to ORASI scale is illustrated in Fig. 6.

## Discussion

Currently the only available pharmacological treatment for OA is non-steroid anti-inflammatory drugs (NSAIDs). Although the use of NSAIDs in osteoarthritis is highly controversial [30], many physicians and patients favor these agents for short and long-term use. However, the therapeutic utility of these agents is frequently limited by the development of side effects especially gastrointestinal ulceration and its complications [31]. Current treatment can control the pain of OA yet side effects could be serious, hence the need to find alternative therapeutic agents. Herbs and extracts present a potential candidate for treatment of OA [5].

In this study we report, for the first time, the beneficial effect of the use of a ginger/curcumin formula in the treatment of OA. Our results show that the ginger/curcumin herbal formula had chondroprotective effect in rat OA model possibly via a combined anti-inflammatory and antioxidant effect.

Our results come in agreement with many researchers who found beneficial effects of ginger and turmeric separately in OA and other related arthritic diseases. Ginger was effective as indomethacin in relieving symptoms of OA with negligible side effects [32]. To our knowledge this is the first report that combines ginger and turmeric extract in one formula as a treatment of OA.

Histopathological evaluation of the ginger/curcumin herbal formula treated groups showed different grades of healing according to Osteoarthritis Research Society International (OARSI) grading system (Fig. 6).

To explain the mechanism of action of ginger/curcumin formula, we measured serum level of COMP as well as other inflammatory and oxidative stress markers in our rat model of OA. Our results show that treatment with herbal formula at two different doses significantly reduced the serum level of COMP compared to OA group (*P* value <0.0001). The herbal formula at dose 400 mg/kg had similar effects on serum COMP levels as Voltaren® as there was a non-significant difference was found between F 400 and V groups (Fig. 1). These results on serum COMP could in part explain the effect of herbal formula observed in our study.

COMP is a large extracellular matrix protein, and a structural component of cartilage. Elevated levels of COMP are released during erosive joint disease such as rheumatoid arthritis and osteoarthritis [33]. The concentration of COMP in OA group was found to be increased over reference levels in the early stages of knee OA development. This is due to series of catabolic events undergoing in articular cartilage, which results in high turnover rate by the chondrocytes in order to repair the cartilage matrix. This process led first to dismantling of cartilage matrix and then a net loss of tissue [34]. There was a significant difference in the concentration of COMP biomarkers between patients with shorter and longer osteophytes on Kondyles of tibia and femur [14]. In another study patients with longer medial osteophytes and with great capsular distension had higher levels of HA and COMP in serum compared to patients with shorter osteophytes [35]. This suggests a role for COMP in the pathogenesis of OA [14].

HA is considered to be a good marker of synovitis in OA as it reflects local inflammation in synovial lining and, cartilage degradation [33]. Our results show that treatment with herbal formula at two-dose levels, significantly reduced serum level of HA compared to OA group (*P* value <0.0001); while its level was significantly increased in OA group compared to control group (Fig. 1).

MPO is released by activated neutrophils and used as a marker of leukocyte recruitment and function and subsequent inflammation. MPO concentration is related to inflammatory activity and could play an important role in the maintenance of oxidative stress in OA [36]. In this study MPO was significantly increased in OA group compared to control group (Fig. 2). On the other hand it was reduced after 1-month treatment with the herbal formula at two-dose levels (*P* value <0.0001).

Increased inflammation is the consequence of many factors, including mechanical overloading, joint injury, adipose tissue, and cartilage matrix fragments. IL-1β is considered one of the most prominent pro-inflammatory cytokines involved in OA. Elevated level of IL-1β is found in OA joint tissues, including the articular cartilage, subchondral bone, synovial fluid and synovium. IL-1β alters the homeostatic balance of chondrocytes by suppressing anabolic activity, stimulating catabolic breakdown of the articular cartilage, and increasing production of inflammatory mediators and reactive oxygen species (ROS) [37].

**Fig. 7:**
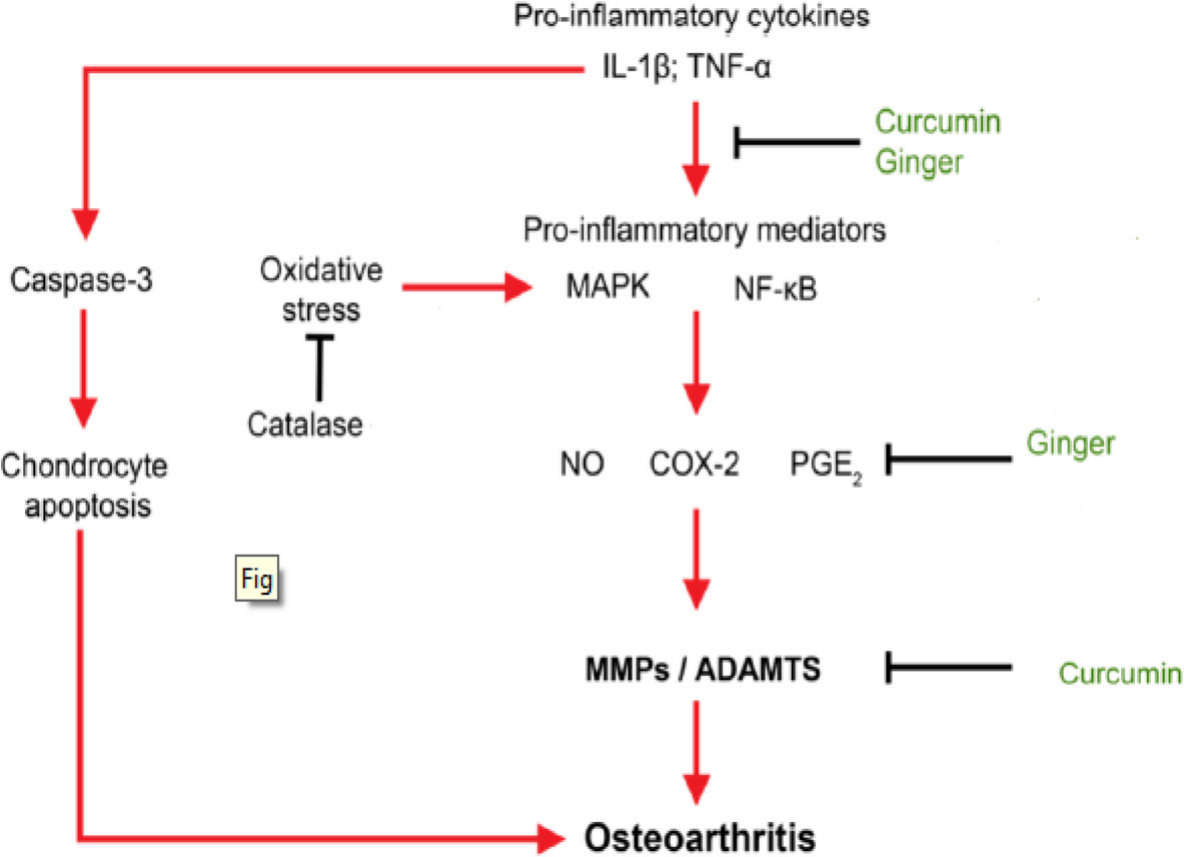
A schematic representation of possible mechanisms by which ginger and curcuma extracts can regulate inflammation level in osteoarthritis. Adapted from Leong et al., 2013 [41]

Ramadan et al., [21] found that the dried rhizomes of turmeric and ginger, separately, reduced inflammation by inhibiting the nuclear factor-κB activation. This lead to inhibition of gene expression of proinflammatory cytokines (TNF-α and IL-1β) in adjuvant-induced rat arthritis. This theory may explain why herbal formula was more effective in reducing inflammation and oxidative stress levels due to the synergistic effects of ginger and turmeric in herbal formula extract (Fig. 7).

Lipid peroxidation may lead to production of several toxic products as MDA. MDA showed a significant decrease in groups treated with herbal formula at two dose levels compared to OA group (*P* value <0.0001). This result comes in agreement with a study that found that there was significant decrease in lipid peroxidation groups treated with *Zingiber officinale* (ginger) when compared to animals treated with DMBA alone. The observed reduction in the level of lipid peroxidation in herbal formula treated animals was presumably due to the ability of ginger to scavenge the hydroxyl and peroxyl radicals [38, 39].

To evaluate the antioxidant effect of the formula we measured serum SOD activity. SOD showed significant elevation in herbal formula treated groups at two dose levels compared to OA group (*P* value <0.0001). This could indicate a direct or indirect (enhancing hepatic antioxidant activity) radical scavenging capability of the formula. Aqueous and ethanolic extract of *Zingiber officinale* was shown to have hepato-protective effect against acetaminophen-induced acute toxicity due to its antioxidant activity [39, 40].

## Conclusion

The composition and characterization of the herbal formula hydro-ethanolic extract confirmed the identification of 11 curcumin and gingerol derivatives. The ginger/curcumin 1:1 herbal formula had chondroprotective effect in rat OA model in rats. Herbal formula treatment also reduced oxidative stress levels. Herbal formula at concentration of 400 mg/kg had more potent anti-inflammatory and anti oxidant effect compared with concentration of 200 mg/kg. Further pharmacological and clinical studies are still needed to evaluate this effect as well as its potential use in treatment of OA in human.

## Abbreviations

COMP: Cartilage oligomeric matrix protein
HA: Hyaluronic acid
IL-1β: Interleukin-1 beta
MDA: Malondialdehyde
MIA: Monosodium Iodoacetate
MPO: Myeloperoxidase
OA: Osteoarthritis
OARSI: Osteoarthritis Research Society International
ROS: Reactive oxygen species
SOD: Superoxide dismutase
